# All-Optical Naked-Eye Ghost Imaging

**DOI:** 10.1038/s41598-020-59263-1

**Published:** 2020-02-12

**Authors:** Gao Wang, Huaibin Zheng, Zhiguo Tang, Yu Zhou, Hui Chen, Jianbin Liu, Yuchen He, Yuan Yuan, Fuli Li, Zhuo Xu

**Affiliations:** 1 Electronic Materials Research Laboratory, Key Laboratory of the Ministry of Education & International Center for Dielectric Research, School of Electronic and Information Engineering, Xi’an Jiaotong University, Xi’an, 710049 China; 2 MOE Key Laboratory for Nonequilibrium Synthesis and Modulation of Condensed Matter, Department of Applied Physics, Xi’an Jiaotong University, Xi’an, 710049 China

**Keywords:** Imaging and sensing, Imaging techniques

## Abstract

Ghost imaging is usually based on the optoelectronic process and electronic computing. A new ghost imaging approach is put forward in the paper that avoids any optoelectronic or electronic process. Instead, the proposed scheme exploits all-optical correlation and the vision persistence effect to generate images observed by naked eyes. To realize high contrast naked-eye ghost imaging, a special pattern-scanning architecture on a low-speed light-modulation disk is designed, which also enables high-resolution imaging with lower-order Hadamard vectors and boosts the imaging speed. With this approach, we realize high-contrast real-time naked-eye ghost imaging for moving colored objects.

## Introduction

Ghost imaging has been an object of research since 1995^1^. Its configuration, usually, consisted of two correlated optical beams propagating in distinct paths and impinging on two spatially-separated photodetectors: the signal beam interacts with an object and then is received by a single-pixel (bucket) detector without spatial resolution, whereas the reference beam goes through an independent path and impinges on a spatial distribution detector (like charge-coupled device, CCD) without interacting with the object. Neither the bucket detector nor CCD can reveal the image of object alone. But, the image can be retrieved by cross-correlating signals from the bucket detector and CCD.

The first ghost imaging experiment was demonstrated by Pittman *et al*.^1^ in 1995 using entangled photon pairs. About ten years later, it was implemented with pseudothermal light^2–5^ and thereafter with true thermal light^6^ as well, such as sunlight. Moreover, computational ghost imaging was introduced by Shapiro to make this imaging technique full of variety, which keeps the signal beam and exploits calculated field pattern rather than the reference beam^7,8^. Due to its novel physical peculiarities, more recent attention has focused on its potential applications in practice. Generally, ghost imaging is applicable to any wavelength and has been recently demonstrated with x-rays^9–12^, atoms^13^, and even electrons^14^. Since ghost imaging can have higher resolution beyond the Rayleigh diffraction limit^15^ and be obtained even in poor illumination^16^ or turbulent atmosphere^17^, it has many potential applications ranging from microscopy^18–20^ to three-dimensional ghost imaging^21^ to long-distance lidar^22,23^ to temporal ghost imaging^24^ and so on.

However, no matter what type of ghost imaging method is, the popular way to get the reconstructed image is by a computer imaging algorithm along with a coincidence measurement (i.e. photoelectric detection process) between the bucket detection process for the signal beam and the known shape of the reference beam. In 2017, a new way to get the reconstructed image with a property of naked-eye ghost imaging^25^ was proposed. Similarly, preliminary work on ghost imaging with the human eye was then undertaken by Boccolini *et al*.^26^, giving its simulation and experiment results. However, from both the theoretical and experimental results at that time, low contrast image is the main obstacle to push this idea closer to practical applications, since the image is immersed in the reference light beam. Recently, we have solved this problem via all-optical process and the persistence of vision with a special pattern-scanning architecture.

In this letter, a high contrast naked-eye ghost imaging by all-optical computation is developed. In this imaging process, the correlated calculation by the photoelectric coincidence measurement of traditional ghost imaging is replaced by an all-optical correlation. While the integral process by the computer imaging algorithm of traditional ghost imaging is implemented by the vision persistence effect, where in principle all photosensitive material with the vision persistence effect can be competent for this integral job. Meanwhile, to solve the slow imaging speed and low contrast image problems, a low-speed light-modulation disk with a special pattern-scanning architecture is proposed, which also enables high-resolution imaging with lower-order Hadamard vectors and boosts the imaging speed. This light-modulation disk is used to generate a series of light patterns and performs the correlated calculation. At last, the imaging system is tested against moving colored objects, and a high contrast image result is observed directly by eyes. Therefore, our work opens a new way to utilize ghost imaging and removes an obstacle to push this idea closer to reality.

## Results

**Figure 1:**
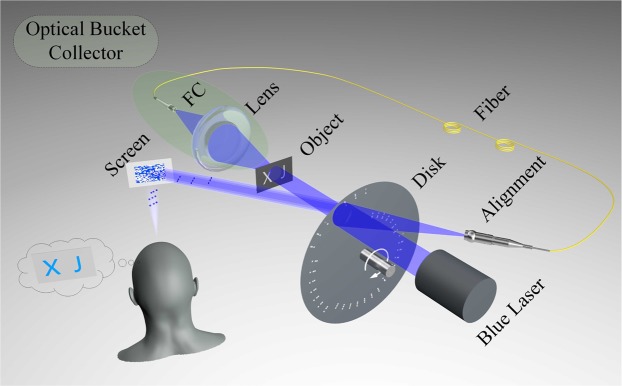
The original experimental scheme for the naked-eye ghost imaging. The high contrast ghost imaging system consists of the all-optical interaction and the vision persistence effect.

The original scheme for the naked-eye ghost imaging is shown in Fig. 1. One blue laser beam is modulated by a rotating light-modulation disk. Then the modulated beam illuminates and interacts with moving objects, that are letters “X”, “J”, “T” and “U” with 35 × 35 pixels, respectively. The transmitted light after the object is collected by a bucket collector setup with a lens and a FC fiber connector. The collected signal beam goes through the fiber and then illuminates the disk overlapping with the blue laser beam area. Note that, two beams propagate in different directions. Here, the correlated calculation is performed by the interaction between the disk and the signal light. Namely, the correlated calculation of the traditional ghost imaging (see Eq. (1)) can be achieved by this setup,

1 $$I(x,y,t)={I}_{1}(x,y,t){I}_{2}(t),$$

where ${I}_{1}(x,y,t)$ stands for the intensity distribution of instantaneous light pattern, ${I}_{2}(t)$ stands for the corresponding bucket value and $I(x,y,t)$ is the multiplication of the correlated calculation. In current setup, the synchronization between ${I}_{1}(x,y,t)$ and ${I}_{2}(t)$ can be realized easily since the light speed is much faster than the rotating speed of the disk. Thus, it is also no need to know the shape of the illuminating pattern and the one-to-one match between each pattern and each bucket value.

The final output light ($I(x,y,t)$) is observed by a photosensitive component such as human eyes, performing the integral process of ghost imaging, that is

2 $${G}^{(2)}(x,y)={\int }_{t}^{t+T}\,I(x,y,t)dt,$$

where *T* stands for the vision persistence time and ${G}^{(2)}(x,y)$ stands for the ghost imaging result. To present the naked-eye ghost imaging result, we use a CCD camera to mimic the vision persistence effect of human eyes. Since the temporary retention time of human eyes is about 0.02 seconds in daytime vision, 0.1 seconds in intermediary vision and 0.2 seconds in night vision, we choose 0.2 seconds as the exposure time of CCD. At this point, a high-contrast real-time imaging will be observed by such photosensitive component once the disk rotating at a rate.

**Figure 2:**
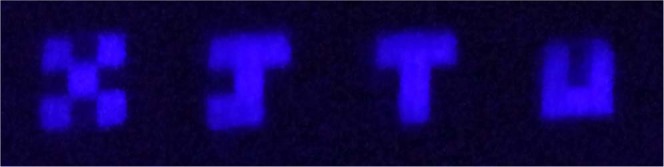
Ghost imaging results via all-optical process and persistence of vision.

Figure 2 shows the high contrast naked-eye ghost imaging results (“X, J, T and U”, respectively). Based on this method, the key problem of the image being immersed in the reference beam is worked around. Meanwhile, the bucket photodetector and the computer algorithm for the typical ghost imaging setup are replaced by the simple all-optical process and the vision persistence effect.

However, current setup has a limitation that the efficiency of the optical bucket collector is very low since the optical fiber coupling is not an easy job. Meanwhile, the lens focal length relies on the light wavelength, which is not suitable for fiber coupling with multi-color situation. And the optical image filtering effect is also introduced. To realize color naked-eye ghost imaging, we improve this setup.

**Figure 3:**
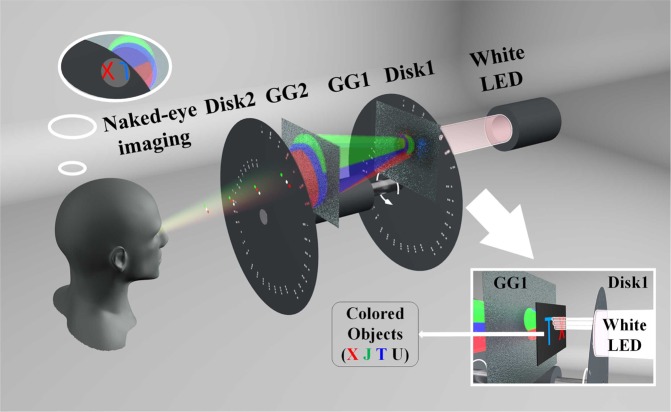
The scheme of the color naked-eye ghost imaging system. GG: ground glass.

The scheme of the high contrast and color naked-eye ghost imaging system is shown in Fig. 3. One white LED beam is modulated by a rotating disk (disk 1, same as mentioned in Fig. 1). Then it illuminates and interacts with colored objects, which are the red letter “X”, the green letter “J”, the blue letter “T” and the white letter “U” with 35 × 35 pixels, respectively. The transmitted light after objects goes through two ground glasses (GG1 and GG2, respectively), which is used to scatter the signal light sufficiently and one can not observe the image directly. Meanwhile, these ground glasses play an optical homogenizer role, which can be understood as a bucket detector in the typical ghost imaging setup. Then, this scatted light propagates through the same arranged disk (disk 2) as disk 1, performing the correlated calculation. Finally, the output light is observed by human eyes, performing the integral process of ghost imaging. Here, two disks are fixed on the same motor, so they are rotating at the same rate. Again, the CCD camera is used to mimic the vision persistence effect of human eyes. By this setup, a high contrast and color image will be observed once two disks rotating at a certain rate.

**Figure 4:**
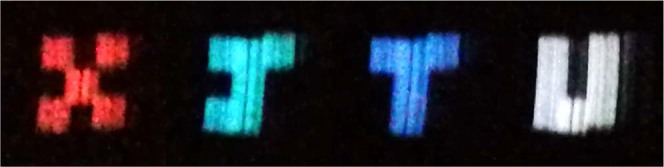
Color naked-eye ghost imaging results via all-optical process and persistence of vision.

Figure 4 shows the high contrast and color naked-eye ghost imaging results (“X, J, T and U”, respectively). In addition, while the reflected light color of “J” is closed to green, the transmitted light color of “J” is almost cyan, contributing to imaging “J” in cyan. Especially, a high-contrast real-time imaging video for moving colored objects is shown in the supplement (see Visualization 1). Based on the improved method, the limitation mentioned above is solved.

Overall, the high-contrast real-time imaging for moving colored objects is realized by such all-optical computation. The bucket detecting process with the photoelectric way and the correlated calculation, as well as the integral process for traditional ghost imaging, are replaced by this new way. The obstacle to realizing this high-contrast real-time imaging for moving colored objects is removed by a special pattern-scanning architecture. Meanwhile, the high resolution and the boosted imaging speed can be obtained with low pixel illumination from a low-speed rotating light-modulation disk.

## Discussion

The current study found that the low-contrast of naked-eye ghost imaging is introduced by the normal pattern structure. So, it is necessary to discuss this influence. For normal ghost imaging process, we define *N* as the pattern pixel number. Ghost imaging can be expressed as follow

3 $${G}^{(2)}=\frac{1}{m}AY\sim AY=A{A}^{T}X\sim CX,$$

where *m* is the numbers of sampling, *A* is the measurement matrix, *Y* is the output value of the bucket detector, *X* is the object. *C* is the autocorrelation matrix of *A*, where the variables *i* and *j* denote the coordinates of the matrix *C*, the *C*_*max*_ and *C*_*min*_ is corresponded to the second moment and the square of the first moment, respectively.

4 $${C}_{ij}=\{\begin{array}{ll}{C}_{{\min }} & i\ne j\\ {C}_{{\max }} & i=j\end{array}.$$

So, one can get the imaging contrast from the definition of Eq. (5), where ${N}_{obj}$ denotes the partial pixel number of the binary image (or objects) except for the background, *I*_*max*_ and *I*_*min*_ denote the max and min value of ghost images. A possible explanation for this might be that the patterns with large value of $\frac{{C}_{ii}}{{C}_{ij}}$ can contribute to high contrast of naked-eye ghost imaging.

5 $$\begin{array}{rcl}Contrast & = & \frac{{I}_{{\max }}-{I}_{{\min }}}{{I}_{{\max }}+{I}_{{\min }}},\\ {I}_{{\max }} & = & {C}_{{\max }}+({N}_{obj}-1){C}_{{\min }},\\ {I}_{{\min }} & = & {N}_{obj}{C}_{{\min }},\end{array},$$

For ghost imaging, getting a suitable light speckle pattern for higher contrast and projecting fewer of speckle pattern in this system remains a challenging task. Usually, the artificially designed light speckle pattern (e.g. Hadamard pattern) is better than the random one, since fewer speckle’s number is needed with the same contrast obtained. Hadamard patterns’ number depends on the total pixel’s number of the object we imaging. Therefore, to get a better imaging result, the way to redesign the structure of Hadamard pattern is a feasible plan.

Hadamard measurement matrix (${N}_{H}\times {N}_{H}$) is a special kind of matrix, in which *N*_*H*_-by-*N*_*H*_ Hadamard matrix with ${N}_{H} > 2$ exists only if the remainder of *N*_*H*_ after division 4 is equal to 0, where *N*_*H*_, *N*_*H*_/12, or *N*_*H*_/20 is a power of 2^27,28^. When one changes the negative matrix elements to zeros for the practical projection, one can figure out that the first pattern and every first pixel of each pattern are 1. It is little significance for projecting the invariant pixels and the speckle with a total of 1 to measure the detail of the object. Not only that, but it will increase the imaging background, leading to a decrease of the imaging contrast. So, it makes sense to abandon these pixels and speckle. Therefore, by abandoning the first row and the first column, the Hadamard measurement matrix will become $({N}_{H}-1)\times ({N}_{H}-1)$. For example, the original Hadamard matrix of order 8 is shown as

6 $$H={[\begin{array}{cccccccc}1 & 1 & 1 & 1 & 1 & 1 & 1 & 1\\ 1 & -1 & 1 & -1 & 1 & -1 & 1 & -1\\ 1 & 1 & -1 & -1 & 1 & 1 & -1 & -1\\ 1 & -1 & -1 & 1 & 1 & -1 & -1 & 1\\ 1 & 1 & 1 & 1 & -1 & -1 & -1 & -1\\ 1 & -1 & 1 & -1 & -1 & 1 & -1 & 1\\ 1 & 1 & -1 & -1 & -1 & -1 & 1 & 1\\ 1 & -1 & -1 & 1 & -1 & 1 & 1 & -1\end{array}]}_{8\times 8}.$$

Then the reduced Hadamard matrix of order 8 becomes

7 $$\hat{H}={[\begin{array}{ccccccc}0 & 1 & 0 & 1 & 0 & 1 & 0\\ 1 & 0 & 0 & 1 & 1 & 0 & 0\\ 0 & 0 & 1 & 1 & 0 & 0 & 1\\ 1 & 1 & 1 & 0 & 0 & 0 & 0\\ 0 & 1 & 0 & 0 & 1 & 0 & 1\\ 1 & 0 & 0 & 0 & 0 & 1 & 1\\ 0 & 0 & 1 & 0 & 1 & 1 & 0\end{array}]}_{7\times 7}.$$

Therefore, one can use 7 patterns reshaped from a 7-row vector of the matrix to get an image. As a result, the operation will contribute to the autocorrelation matrix *C*_*ij*_ as

8 $${C}_{ij}=\{\begin{array}{ll}1 & i\ne j\\ 3 & i=j\end{array}.$$

And the background will be one third of the peak when using the small hole as an object. The contrast is up to 1/2. In this way, the relationship between contrast and resolution is obtained. One can get the expression of *C*_*ij*_

9 $${C}_{ij}=\{\begin{array}{ll}\frac{N+1}{4}-1 & i\ne j\\ \frac{N+1}{2}-1 & i=j\end{array},$$

where $N+1$ is the order (*N*_*H*_) of the Hadamard matrix, and the pattern pixel’s number is *N* with $N\ge 3$. When taking Eqs. (5) and (9) into account, Eq. (10) can be obtained,

10 $$contras{t}_{\hat{H}}=\frac{1+N}{1+N+2{N}_{obj}(N-3)}.$$

From Eq. (10), one can get that *contrast*_*Ĥ*_ increases with decreasing *N*_*obj*_. So, this method can improve the contrast ratio of small duty ratio area imaging. Moreover, the pattern reshaped from the reduction Hadamard matrix will remain only one lighted pixel when $N=3$, which is similar to the laser point scanning technique.

## Methods

In the method section, we will show how to realize this high contrast and color naked-eye ghost imaging process. In the whole idea, the key component is the specially designed pattern-scanning architecture on a low-speed light-modulation disk. It realizes two functions: Firstly, it is used to generate a series of the light pattern and does the correlated calculation. Secondly, its special design ensures the high contrast imaging results. Now we show this idea step by step.

### Step 1: Imaging partition in the first dimension

**Figure 5:**
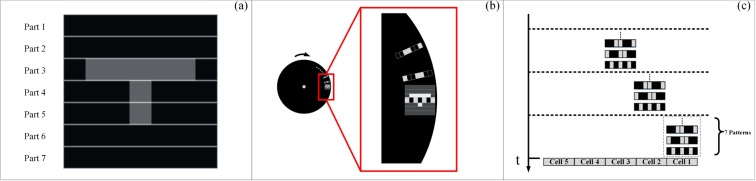
(**a**) Imaging partition in the first dimension. (**b**) A rotating mask with a pattern hole. (**c**) Cell structure.

As shown in Fig. 5(a), the object (*n* × *n*) is divided into *n* row part with each part is 1 × *n*. It can increase local contrast in imaging, and the effect of detail enhancement is achieved. Here, the part contrast is

11 $$\begin{array}{rcl}contras{t}_{\hat{H}\_Part} & = & \frac{1+{N}_{Part}}{1+{N}_{Part}+2{N}_{obj}({N}_{Part}-3)},\\ {N}_{Part} & = & \sqrt{N}=n.\end{array}$$

Generally, digital micromirror device (DMD) can realize this work. One can project the 1 × *n* Hadamard pattern *n* times to the row part of the object for this part imaging, step by step. When the last row part of the object is finished, the imaging result can get.

### Step 2: New projection method

The projection method is easy to understand. However, we can change the projection order that one can use the first 1 × *n* Hadamard vector to go through each row part of the object from top to bottom. Then, use the remaining 1 × *n* Hadamard vectors do the same thing. We call it as pattern moving. So, one can improve the resolution by reducing the step length. In detail, the new projecting method is realized by a rotating mask with a pattern hole on the edge, as shown in Fig. 5(b). It is a low-cost device that can be formed very quickly. In addition, there are *n* mask to imaging *n* × *n* object. This method can greatly reduce the number of mask.

### Step 3: Imaging partition in the second dimension

In order to get a higher contrast, one need do the imaging partition in the second dimension. One can divide the row part object (1 × *n*) into *k* row cell with each small cell be 1 × (*n*/*k*) as shown in Fig. 5(c). Instead of using the 1 × *n* Hadamard pattern moving, we use the Hadamard cell moving, which consists of a complete set of Hadamard pattern. Its contrast can be expressed as

12 $$\begin{array}{rcl}contras{t}_{\hat{H}\_Cell} & = & \frac{1+{N}_{Cell}}{1+{N}_{Cell}(2{N}_{Cell}-5)},\\ {N}_{Cell} & = & \frac{\sqrt{N}}{k}.\end{array}$$

In order to get high contrast via Hadamard pattern, apart from the sample scanning, it is a suitable choice that one can take ${N}_{Cell}=7$. So, we adopt $n=35$, $k=5$, and ${N}_{Cell}=7$ to realize imaging.

## Supplementary information

Supplementary Information.
